# Defect Engineering in Fluorinated Metal–Organic Frameworks Within Mixed-Matrix Membranes for Enhanced CO_2_ Separation

**DOI:** 10.3390/membranes15100296

**Published:** 2025-09-30

**Authors:** Benxing Li, Lei Wang, Yizheng Tao, Rujing Hou, Yichang Pan

**Affiliations:** State Key Laboratory of Materials-Oriented Chemical Engineering, College of Chemical Engineering, Nanjing Tech University, Nanjing 210009, China; 202461104013@njtech.edu.cn (B.L.); 202362042066@njtech.edu.cn (L.W.); 202361204444@njtech.edu.cn (Y.T.); rujing.hou@njtech.edu.cn (R.H.)

**Keywords:** mixed-matrix membranes, fluorinated MOFs, defect engineering, CO_2_ separation

## Abstract

Developing highly permeable and selective membranes for energy-efficient CO_2_/CH_4_ separation remains challenging. Mixed-matrix membranes (MMMs) integrating polymer matrices with metal–organic frameworks (MOFs) offer significant potential. However, rational filler–matrix matching presents substantial difficulties, constraining separation performance. In this work, defects were engineered within fluorinated MOF ZU-61 through the partial replacement of 4,4′-bipyridine linkers with pyridine modulators, producing high-porosity HP-ZU-61 nanoparticles exhibiting a 267% BET surface area enhancement (992.9 m^2^ g^−1^) over low-porosity ZU-61 (LP-ZU-61) (372.2 m^2^ g^−1^). The HP-ZU-61/6FDA-DAM MMMs (30 wt.%) demonstrated homogeneous filler dispersion and pre-served crystallinity, achieving a CO_2_ permeability of 1626 barrer and CO_2_/CH_4_ selectivity (33), surpassing the 2008 Robeson upper bound. Solution-diffusion modeling indicated ligand deficiencies generated accelerated diffusion pathways, while defect-induced unsaturated metal sites functioned as strong CO_2_ adsorption centers that maintained solubility selectivity. This study establishes defect engineering in fluorinated MOF-based MMMs as a practical strategy to concurrently overcome the permeability–selectivity trade-off for efficient CO_2_ capture.

## 1. Introduction

Natural gas and biomethane as clean energy sources consist mainly of methane and carbon dioxide. However, CO_2_ significantly reduces the calorific value of natural gas, increases transportation costs, and, critically, exhibits corrosivity in humid environments, causing severe pipeline/equipment damage in water [1,2]. Consequently, CO_2_ must be removed to <2 mol% prior to long-distance transportation or liquefied natural gas plant processing. Membrane-based separation stands out as an energy-saving alternative to traditional thermal methods such as distillation, adsorption, and absorption for CO_2_ capture, offering significant potential to enhance energy efficiency while reducing costs and carbon intensity [3,4]. However, further improvement of CO_2_ separation efficiency to enhance the competitiveness of membrane separation remains challenging owing to the intrinsic permeability–selectivity trade-off of traditional polymeric membrane materials [5].

As composites of processable polymer matrices and molecular-sieving inorganic fillers, mixed-matrix membranes represent an effective approach to enhance separation efficiency by integrating complementary advantages of both phases [6]. Owing to their unprecedented tunability in pore size, porosity, topologies, dimensions, and chemical functionalities, metal–organic frameworks (MOFs) have emerged as a highly promising filler material for fabricating high-performance MMMs tailored to specific separation applications [7]. Although MOF/polymer composites provide multiple optimization pathways for advanced membrane materials, filler–matrix incompatibility [8,9], MOF particle agglomeration/sedimentation [10], and polymer–MOF permeability mismatch collectively impede the simultaneous enhancement of gas permeability and selectivity to overcome the intrinsic trade-off limitation [5].

Metal–organic frameworks are constructed by connecting metal clusters with organic linkers via strong coordination bonds, forming ordered porous crystalline structures [11]. Conventionally, pristine MOF crystals with defect-free frameworks were considered optimal MMMs fillers to maximize their molecular sieving capabilities. Intriguingly, deliberate introduction of defects into MOF architectures demonstrated unexpected efficiency enhancements, including elevated adsorption capacities, boosted catalytic activity, and enhanced electronic conductivity, attributable to the engineered porosity and abundant active sites [12]. A paradigmatic example is defect-engineered UiO-66, where controlled missing-linker defects are quantitatively incorporated using monocarboxylate modulators during synthesis. Given the superior gas adsorption of defective MOFs, mixed-matrix membranes incorporating such materials are anticipated to deliver significantly enhanced gas separation performance [13].

**Figure 1:**
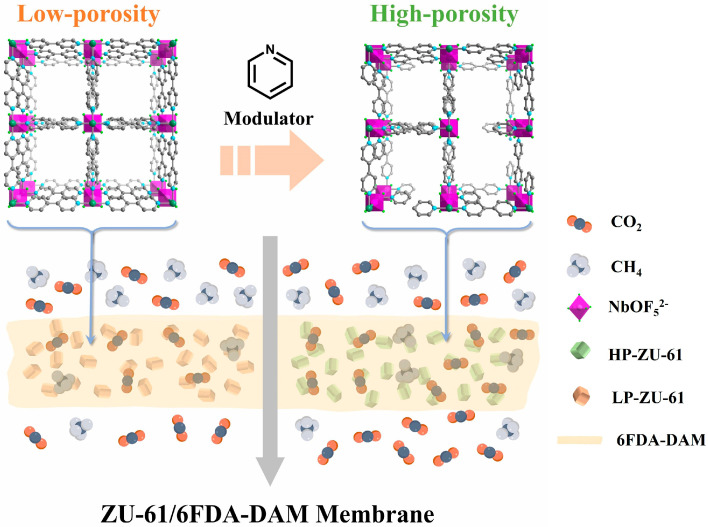
Schematic comparing defect-engineered LP-ZU-61 and HP-ZU-61 fillers in 6FDA-DAM polymer: LP-ZU-61 improves selectivity, whereas HP-ZU-61 achieves synergistic CO_2_ permeability/selectivity enhancement.

Inspired by this strategy, defect engineering was implemented in ZU-61 (ZU = Zhejiang University, also termed NbOFFIVE-bpy-Ni, NbOFFIVE = NbO_5_^2−^, bpy = 4,4′-bipyridine) crystals using pyridinic modulators (Figure 1). This fluorinated MOF features 2D grids pillared by (NbOF_5_)^2−^ anions, where periodically aligned (NbOF_5_)^2−^ arrays within 1D channels generate specific CO_2_-philic electrostatic interactions, conferring exceptional CO_2_ adsorption performance. To decouple porosity effects from other variables, two ZU-61 nanoparticles with a comparable particle size, morphology, and colloidal stability, but contrasting porosities, were synthesized as MMM fillers. Material characterization confirmed nearly identical filler–matrix interface characteristics between high-porosity ZU-61 (HP-ZU-61) and low-porosity ZU-61 (HP-ZU-61)-based MMMs. Crucially, HP-ZU-61 MMMs demonstrated simultaneous enhancement of CO_2_ permeability and CO_2_/CH_4_ selectivity, surpassing the 2008 Robeson upper bound. Conversely, LP-ZU-61/6FDA-DAM MMMs only improved selectivity. This performance divergence unequivocally attributes the separation enhancement to defect-induced superiority, validating the critical role of MOF defects in optimizing the gas transport properties of MMMs.

## 2. Experimental Section

### 2.1. Materials

Nickel(II) nitrate hexahydrate (Ni(NO_3_)_2_·6H_2_O, 97%) and niobium(V) oxide (Nb_2_O_5_) were sourced from Sigma-Aldrich (Shanghai, China) Trading Co., Ltd. The 3-Methylpyridine (C_6_H_7_N, 99%) and 4,4′-bipyridine (C_10_H_8_N_2_, 98%) were obtained from Sigma-Aldrich. The 4,4′-(Hexafluoroisopropylidene) diphthalic anhydride (6FDA, C_19_H_6_F_6_O_6_, 99%) and 2,4,6-trimethyl-1,3-phenylenediamine (DAM, C_9_H_14_N_2_, 96%) were purchased from Shanghai Macklin Biochemical Technology Co., Ltd. (Shanghai, China) Hydrofluoric acid (HF), methanol (MeOH), chloroform (CHCl_3_), acetic anhydride (C_4_H_6_O_3_), and ethylene glycol ((CH_2_OH)_2_) were supplied by Sinopharm Chemical Reagent Co., Ltd. Gases (Air, He, N_2_, CO_2_, and CH_4_) were provided by Nanjing Special Gas Factory Co., Ltd. (Nanjing, China). Deionized (DI) water was obtained from laboratory purification systems.

### 2.2. Synthesis of ZU-61 Nanoparticles

ZU-61 nanoparticles were synthesized via a modulated solvothermal method. In a typical procedure, 0.3124 g (2 mmol) of 4,4′-bipyridine (main ligand) was dissolved in 20 mL of an ethylene glycol/water mixture (19:1 *v/v*) in an autoclave. Then, 0.3 mL of NiNbOF_5_ solution (0.5 mmol, 1.680 mol·L^−1^) was added under vigorous stirring. For LP-ZU-61 (low porosity), the reaction proceeded without any modulator for 20 min at room temperature before sealing the autoclave. For HP-ZU-61 (high porosity), 1 mmol of pyridine modulator was introduced into the mixture after the addition of NiNbOF_5_, followed by stirring for 20 min. Both reaction mixtures were then heated at 80 °C for 24 h. The resulting crystalline products were isolated by high-speed centrifugation, thoroughly rinsed with methanol three times (10 mL per wash), and redispersed in chloroform for storage.

### 2.3. Synthesis of 6FDA-DAM

The 6FDA-DAM polyimide was synthesized via a two-step polycondensation using equimolar amounts of dianhydride (6FDA) and diamine (DAM) monomers. Specifically, 1.5022 g (10 mmol) DAM was dissolved in DMAc, followed by addition of 4.4424 g (10 mmol) 6FDA powder under N_2_ atmosphere. The total monomer concentration was maintained at 20 wt.% during 24 h reaction at 0–5 °C, yielding a highly viscous PAA solution. Subsequently, dehydrating agent acetic anhydride (40 mmol) and catalyst triethylamine (10 mmol) (4:1 molar ratio) were introduced to trigger imidization at ambient temperature for 24 h. The resulting polyimide solution was precipitated into excess methanol, and the purified polymer was vacuum-dried at 150 °C for 12 h.

### 2.4. Preparation of ZU-61/6FDA-DAM MMMs

MMMs with varying MOF loadings were prepared via solution casting. First, 6FDA-DAM polymer was vacuum-dried overnight at 100 °C to remove residual solvents and moisture. Subsequently, 0.3 g polymer was dissolved in 8 mL CHCl_3_ and filtered. Pre-synthesized MOF dispersions (35 mg/mL HP-ZU-61 and LP-ZU-61 in CHCl_3_) were added at MOF/(MOF + polymer) mass ratios of 0, 10, 20, and 30 wt.% (corresponding to 0 g, 0.95 g, 2.4 g, and 3.7 g dispersion, respectively). Each mixture was adjusted to 4 g total mass with additional CHCl_3_ before combining with the polymer solution. The blends were roll-mixed for 12 h, degassed under vacuum for 30 min to eliminate micro-bubbles, and homogenized by ultrasonication (5 min). The solutions were then cast onto glass Petri dishes, stored in sealed bags for 48 h, and vacuum-annealed at 120 °C for 12 h. Membrane thickness was measured using a Mitutoyo digital micrometer (Japan).

### 2.5. Gas Permeation Measurements

Gas permeation properties of MMMs were evaluated using a Wicke–Kallenbach apparatus, shown in Figure S1. Membranes were tested at 298 K and 2 bar feed pressure with an equimolar CO_2_/CH_4_ mixture (total flow rate: 50 mL·min^−1^). A 50 mL·min^−1^ helium sweep stream minimized concentration polarization. Upon reaching steady-state conditions, the permeate stream composition was analyzed by gas chromatography (Agilent 7890B, Agilent Technologies, Santa Clara, CA, USA) equipped with a thermal conductivity detector (TCD). Reported gas permeabilities represent averages from three independently fabricated membrane replicates. The permeability $P_{i}$ (barrer, 1 barrer = 10^−10^ cm^3^(STP)·cm·cm^−2^·s^−1^·cmHg^−1^) is defined by Equation (1):

(1) $$P_{i}=\frac{Q_{i}l}{A∆P}$$

where $Q_{i}$ (cm^3^(STP)·s^−1^) denotes the standard volumetric flux, l (cm) is the membrane thickness, A (cm^2^) represents the effective membrane area, and ∆P (cmHg) is the transmembrane pressure difference. Gas permeation properties were evaluated using a Wicke–Kallenbach apparatus. The separation factor $\alpha_{ij}$ for the equimolar CO_2_/CH_4_ gas mixture was determined experimentally based on the compositions of the permeate and retentate sides under steady-state conditions, as defined by Equation (2):

(2) $$\alpha_{ij}=\frac{P_{i}}{P_{j}}=\frac{{\left(\frac{y_{i}}{y_{j}}\right)}_{p}}{({\frac{x_{i}}{x_{j}})}_{r}}$$

where x and y denote the retentate and permeate concentrations, respectively. According to the solution-diffusion mechanism, the permeability of a gas component through the membrane is defined as the product of its solubility coefficient (S) and diffusion coefficient (D). Thus, the separation factor (α) is governed by the product of solubility selectivity ($\alpha_{S}$) and diffusivity selectivity ($\alpha_{D}$):

(3) P=S·D

(4) $$\alpha_{ij}=\frac{P_{i}}{P_{j}}=\frac{S_{i}}{S_{j}}\cdot \frac{D_{i}}{D_{j}}=\alpha_{S}\cdot \alpha_{D}$$

In Equation (4), $S_{i}$ and $S_{j}$ (cm^3^(STP)·cm^−3^·cmHg^−1^) denote the solubility coefficients of gas components i and j, respectively, while $D_{i}$ and $D_{j}$ (cm^2^·s^−1^) represent their diffusion coefficients. Adsorption coefficients can be independently determined via pressure-decay adsorption and are expressed.

(5) $$S=\frac{c}{f}$$

where c (cm^3^(STP)·cm^−3^) is the concentration of the adsorbed component in the sample and f (cmHg) represents the corresponding upstream fugacity driving force. The dual-mode sorption model was employed to analyze gas adsorption concentrations in glassy polymeric membranes, as described by Equation (5):

(6) $$c=c_{d}+c_{h}=k_{d}\cdot f+\frac{c_{h}^{\prime }\cdot b\cdot f}{1+b\cdot f}$$

where $c_{d}$ (cm^3^(STP)·cm^−3^) denotes the dissolved gas concentration governed by Henry’s law, $c_{h}$ (cm^3^(STP)·cm^−3^) represents the adsorbed gas concentration following Langmuir isotherm behavior, $k_{d}$ (cm^3^(STP)·cm^−3^·cmHg^−1^) is the Henry’s law solubility coefficient, $c_{h}^{\prime }$ (cm^3^(STP)·cm^−3^) signifies the Langmuir saturation constant, and b (cmHg^−1^) indicates the Langmuir affinity constant [12].

The diffusion coefficient D is given by the ratio of permeability to solubility coefficient [12]:

(7) $$D=\frac{P}{S}$$

### 2.6. Characterization

Chemical compositions of ZU-61 particles were analyzed by ^1^HNMR spectroscopy (400 MHz, Mercury Vx) dissolved in D_2_O. Crystal structures of MOFs and membranes were characterized by X-ray diffraction (XRD, Rigaku Smartlab TM 9 kW, Rigaku Corporation, Tokyo, Japan). Chemical environments were probed using attenuated total reflectance Fourier-transform infrared spectroscopy (ATR-FTIR, Nicolet iS10, Thermo Fisher Scientific, Waltham, MA, USA). Crystal morphologies of MOFs and MMM interfacial compatibilities were investigated by field-emission scanning electron microscopy (FE-SEM, Hitachi S4800, Hitachi High-Tech, Tokyo, Japan). Thermal stability of MOFs was evaluated via synchronous thermal analysis (NETZSCH STA 449, NETZSCH Group, Selby, Germany) under N_2_ flow (10 °C min^−1^, 25–800 °C). N_2_ adsorption–desorption isotherms at 77 K were recorded on a BSD-660M analyzer, with BET surface areas calculated from adsorption branches. CO_2_, CH_4_, and N_2_ adsorption capacities at 298 K and 1 atm were determined for both MOFs and membranes. Prior to measurements, samples (50 mg) were degassed at 150 °C under N_2_ for 12 h.

## 3. Results and Discussion

**Figure 2:**
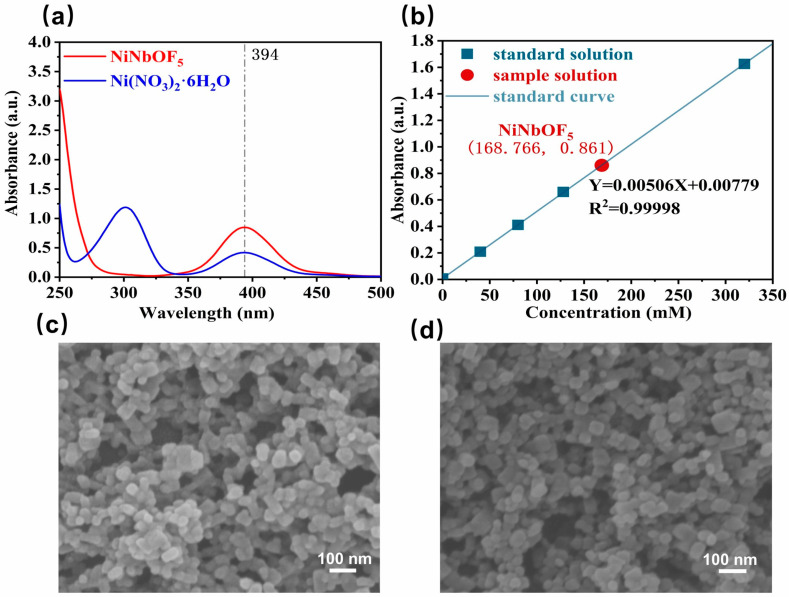
(**a**) UV–Vis spectrum of NiNbOF_5_ solution and Ni(NO_3_)_2_·6H_2_O solution; (**b**) calibration curve for NiNbOF_5_ solution quantification; SEM images of (**c**) HP-ZU-61 and (**d**) LP-ZU-61 nanoparticles.

Fluorinated MOFs such as ZU-61 are conventionally synthesized via a one-pot solvothermal reaction of Nb_2_O_5_, Ni(NO_3_)_2_·6H_2_O, HF, and 4,4′-bipyridine. While this method is operationally straightforward, it requires control of multivariable optimization and involves hazardous concentrated HF. We therefore adopted a stepwise synthesis involving first preparing the NiNbOF_5_ intermediate followed by its reaction with ligands to obtain ZU-61 crystals [14]. This approach reduces the number of reactant variables from 4 to 2, enabling precise control over the crystal morphology and pore architecture. Crucially, unlike prior protocols, the heating of NbOF_5_^2-^ ions results in the formation of discernible amounts of NbO_2_F (observed as a white powder), an undesirable byproduct. The NiNbOF_5_ solution concentration was determined by UV–Vis spectroscopy, with Ni^2+^ exhibiting its maximum absorbance at 394 nm (Figure 2a). A linear calibration curve was established using aqueous Ni(NO_3_)_2_·6H_2_O standards, enabling quantification of the NiNbOF_5_ solution concentration as 1.680 mol·L^−1^ (Figure 2b).

Nano-scale uniform crystallites are recognized as the optimal filler morphology for mixed-matrix membranes [15]. While the morphology of ZU-61 is critically dependent on the reaction solvent’s composition, as shown in Figure S2, ZU-61 particles with distinct morphologies were synthesized by modulating the ratio of ethylene glycol to water in the reaction solvent. When the water content in the solvent was higher than 40 vol%, the XRD patterns of the synthesized particles deviated significantly from the simulated ZU-61 profile (Figure S3). When the content of ethylene glycol in the solvent was higher than 60 vol%, crystallite homogeneity and monodispersity were significantly improved, and the crystal size was reduced to about 50 nm (Figure S2d–f). Phase-pure ZU-61 nanoparticles were consistently obtained under low-water conditions, as confirmed by the peak-to-peak consistency between the experimental and simulated diffraction patterns (Figure S3).

**Figure 3:**
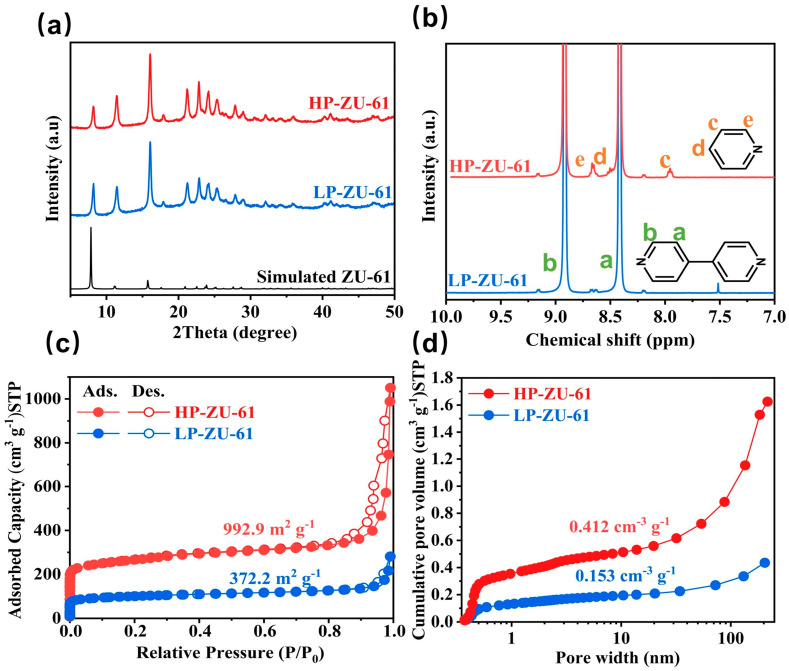
(**a**) XRD patterns of HP-ZU-61 and LP-ZU-61; (**b**) ^1^H NMR of HP-ZU-61 and LP-ZU-61; (**c**) N_2_ sorption isotherms of HP-ZU-61 and LP-ZU-61 particles at 77K; (**d**) pore size distributions of HP-ZU-61 and LP-ZU-61.

Defect-engineered, high-porosity ZU-61 nanoparticles were prepared by introducing pyridine as a modulator into the precursor mixture to partially replace the 4,4′-bipyridine organic ligands, thereby creating lattice defects. As shown in Figure 2c,d, there is no significant difference in the morphology and size of ZU-61 particles with high and low porosity, which circumvents the performance bias due to the filler morphology for the subsequent membrane performance studies. In addition, XRD patterns of a series of ZU-61 samples revealed that all diffraction peaks of the above samples matched well with the standard ZU-61 phase, which indicates that the addition of the pyridine modifier did not cause any damage to the original backbone structure of ZU-61 (Figure 3a and Figure S5a). The FTIR spectra (Figure S4a) reveal essentially identical characteristic absorption bands for HP-ZU-61 and LP-ZU-61, with bands at 1607 cm^−1^ and 922 cm^−1^ corresponding to C-N bond stretching vibrations and Nb-O stretching vibrations in (NbOF_5_)^2-^, respectively [16,17]. Thermogravimetric analysis (TGA) under a nitrogen atmosphere revealed nearly identical thermogravimetric profiles for both materials (Figure S4b). The ^1^H NMR spectra of LP-ZU-61 display proton peaks in the intermediate and neighboring positions of 4,4′-bipyridine at 8.45 ppm (a) and 8.95 ppm (b). In addition, the ^1^H NMR pattern of HP-ZU-61 shows three new distinct proton peaks (c, d, and e), which originated from the protons at the interstitial, para, and neighboring positions of the pyridine, respectively (Figure 3b). This demonstrates that the pyridine modulator participates in the formation of the ZU-61 framework through partial replacement of the bipyridine linkers.

To systematically elucidate the non-linear correlation between modulator concentration and porosity, a series of ZU-61 samples were synthesized with progressively increasing pyridine amounts (0, 0.5, 1.0, and 1.5 mmol). N_2_ adsorption–desorption isotherms measured at 77 K (Figure 3c and Figure S5b) exhibited a distinct bell-shaped trend: the BET specific surface area increased progressively from 372.2 m^2^ g^−1^ for the low-defect LP-ZU-61 (0 mmol pyridine) to 549.2 m^2^ g^−1^ (0.5 mmol), reaching a maximum of 992.9 m^2^ g^−1^ for the HP-ZU-61 (1.0 mmol pyridine). This improvement is directly correlated with the introduction of a higher density of missing-linker defects. However, further increasing the pyridine concentration to 1.5 mmol resulted in a pronounced decrease in surface area to 634.1 m^2^ g^−1^. This reduction is likely due to partial framework degradation at excessive modulator loadings, potentially leading to disordered pore structures and compromised long-range crystallinity. Therefore, an optimal modulator concentration is critical in order to maximize porosity while maintaining structural integrity. Based on these results, the 1.0 mmol pyridine condition (HP-ZU-61) was selected for subsequent membrane fabrication and comparative evaluation with LP-ZU-61 (0 mmol) to clearly demonstrate the effect of optimized defect engineering on gas separation performance. The cumulative volume calculated by density functional theory (DFT) indicated that the total pore volume increased from 0.435 cm^3^ g^−1^ to 1.625 cm^3^ g^−1^ [13]. Crucially, there was also a significant difference in the pore volume of micropores below 2 nm between the low- and high-porosity samples, which were 0.153 cm^3^ g^−1^ and 0.412 cm^3^ g^−1^ (Figure 3d). The disparity in microporous and mesoporous pore volumes strongly indicates that high-porosity ZU-61 incorporates not only ligand deficiency but also maybe metal-node defects with its framework [18]. Analysis of the pore size distribution (Figure S6) reveals that the primary micropore peak remains largely consistent between LP-ZU-61 and HP-ZU-61, centered around 0.6 nm. This demonstrates that the defect-engineering strategy utilizing a pyridine modulator predominantly generates additional porosity without substantially modifying the intrinsic pore aperture size responsible for molecular sieving.

**Figure 4:**
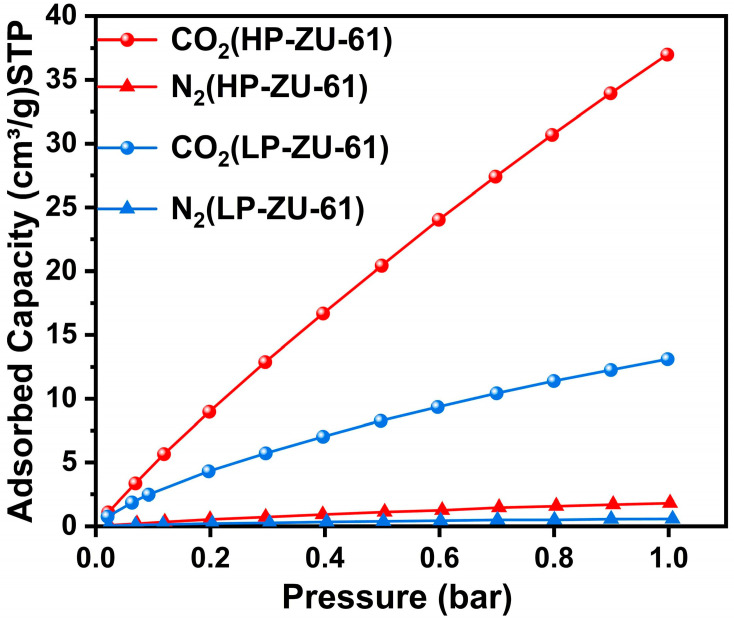
CO_2_, N_2_ adsorption isotherms of HP-ZU-61 and LP-ZU-61 particles at 25 °C.

Volumetric adsorption analysis at 298 K and 1 atm (Figure 4) quantitatively demonstrates the superior CO_2_ affinity of defect-engineered HP-ZU-61 nanoparticles over pristine LP-ZU-61, with CO_2_ uptake increased by 300% at 1 bar (37.52 vs. 12.47 cm^3^ g^−1^). Notably, the N_2_ adsorption capacity concurrently rose by 143.75% (0.23 vs. 0.16 cm^3^ g^−1^), indicating a modulated affinity toward quadrupolar molecules. This marked adsorption enhancement originates from two synergistic effects within defect-tailored frameworks: (i) unsaturated metal centers (Lewis acid sites) generated by ligand deficiencies strengthen electrostatic interactions with CO_2_ carbonyl oxygen and (ii) engineered mesopores (2–5 nm range) facilitate multilayer adsorption via reduced diffusion barriers. Dual-mode sorption modeling (Table S1) further corroborates these mechanisms, revealing a 256% higher Langmuir adsorption capacity for CO_2_ in HP-ZU-61 (33.48 vs. 13.08 cm^3^·cm^−3^ in LP-ZU-61), which directly translates to an enhanced solubility coefficient in HP-ZU-61-based MMMs.

Interfacial compatibility of the filler–matrix remains a critical prerequisite for high-performance mixed-matrix membranes [19]. Incompatible filler matrices inevitably generate nonselective interfacial defects, degrading membrane selectivity. 6FDA-DAM polyimide is a benchmark polymer matrix with CO_2_/CH_4_ separation performance approaching the 2008 Robeson upper bound [20], while MOF/6FDA-DAM MMMs consistently exhibit superior separation characteristics (Table S4).

**Figure 5:**
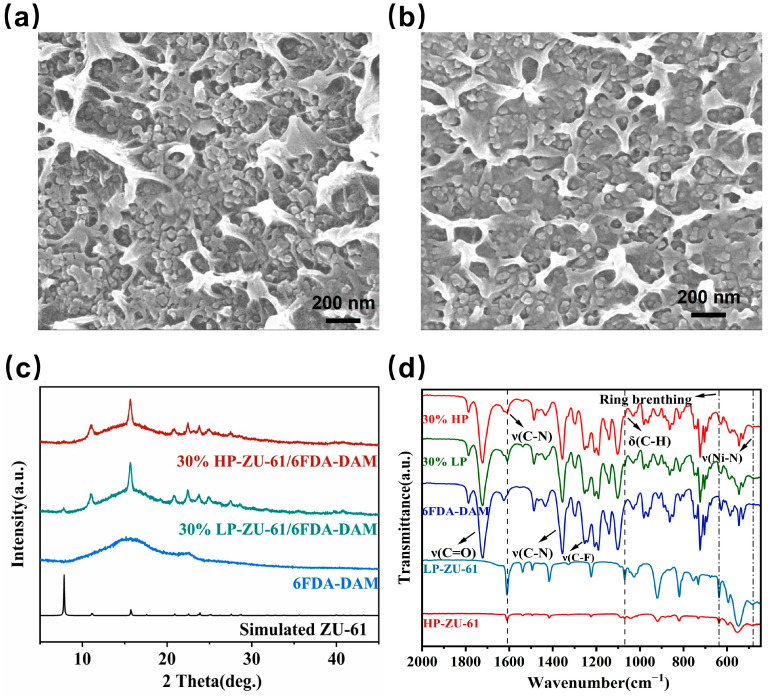
Cross-sectional SEM images of (**a**) HP-ZU-61/6FDA-DAM membrane with loading of 30 wt.%; (**b**) LP-ZU-61/6FDA-DAM membrane with loading of 30 wt.%; (**c**) XRD patterns of 6FDA-DAM and ZU-61/6FDA-DAM membranes with loading of 30 wt.%; (**d**) FTIR of HP-ZU-61 nanoparticles, LP-ZU-61 nanoparticles, 6FDA-DAM membrane, 30% LP (30 wt.% LP-ZU-61/6FDA-DAM membrane) and 30% HP (30 wt.% HP-ZU-61/6FDA-DAM membrane).

We explored the possible formation of interfacial voids in ZU-61/6FDA-DAM MMMs prepared by the solution casting method. Cross-sectional SEM images (Figure 5a,b) reveal that the ZU-61 nanoparticles are homogeneously dispersed within the polymer matrices, with no interfacial voids observed. This exceptional compatibility is attributed to: (i) sub-50 nm crystallites and (ii) hydrogen-bonding interactions between ligand hydrogen atoms and carbonyl groups of 6FDA-DAM to enhance compatibility with the polymer [21]. Crucially, the near-identical interfacial characteristics enable the attribution of the divergent gas transport behavior solely to the intrinsic properties of the MOF, providing definitive evidence for the observed selectivity enhancement.

The XRD analysis (Figure 5c) quantitatively confirmed the preservation of ZU-61 crystallinity within MMMs, as evidenced by characteristic diffraction peaks at 2θ = 8.5°, 12.1°, and 17.3°. This exceptional structural integrity demonstrates negligible framework distortion during membrane processing despite polymer chain infiltration. Crucially, the retained crystallographic order ensures unaltered molecular sieving functionality, which directly contributes to the observed 33.4 CO_2_/CH_4_ selectivity in 30 wt.% HP-ZU-61/6FDA-DAM membranes. The absence of discernible peak broadening conclusively excludes significant MOF amorphization or interfacial stress-induced defects. These structural preservation characteristics are critical for maintaining a stable separation performance during an extended 72 h of continuous operation [22].

The microstructure of MMMs was analyzed by ATR-FTIR. The infrared spectra (Figure 5d) revealed that the characteristic bands of 6FDA-DAM were identified in the 30 wt.% HP-ZU-61/6FDA-DAM membrane (C=O:1721 cm^−1^, C-F:1250 cm^−1^). The corresponding spectra of pure polymer membranes are in agreement with the literature reports [23,24]. The successful integration of ZU-61 nanoparticles within the polymer matrix is unambiguously confirmed by the emergence of characteristic vibrational modes associated with the filler in the FTIR spectra of the MMMs (Figure 5d). A direct comparison between the spectra of pristine nanoparticles of HP-ZU-61 and LP-ZU-61 and that of the nanoparticle-filled MMMs demonstrated the consistent behavior of the C-N stretching vibration at 1607 cm^−1^, the C-H bending vibration of 4,4′-bipyridine at 1090 cm^−1^, and the Ni-N stretching vibration at 430 cm^−1^ [16,21] without noticeable broadening or shifting. Such performance indicates that the fillers were successfully incorporated into the polymer matrix. A distinct ring-breathing mode at 680 cm^−1^ was observed in all MMMs [16]. The relative intensity variations of this band correlate directly with pyridine modulator-induced framework defects (as validated by ^1^H NMR), thus altering the ligand ring symmetry vibration mode. These vibrational signatures collectively confirm the successful construction of defect-engineered HP-ZU-61 nanoparticles and their effective integration within the 6FDA-DAM matrix.

**Figure 6:**
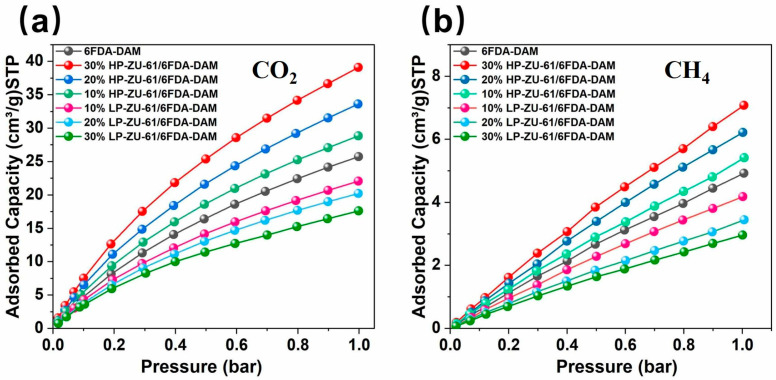
(**a**) CO_2_ adsorption isotherms of (10 wt.%–30 wt.%) ZU-61/6FDA-DAM and 6FDA-DAM membranes at 298 K; (**b**) CH_4_ adsorption isotherms of (10 wt.%–30wt.%) ZU-61/6FDA-DAM and 6FDA-DAM membranes at 298 K.

Defect engineering in regulating CO_2_ adsorption within ZU-61/6FDA-DAM MMMs was investigated. Ambient-pressure volumetric adsorption analysis was employed. This quantified the CO_2_ uptake behaviors of MMMs containing 10–30 wt.% ZU-61. As shown in Figure 6a, CO_2_ adsorption was systematically enhanced with increasing HP-ZU-61 nanoparticle loading. Notably, 30 wt.% HP-ZU-61/6FDA-DAM MMMs exhibit a 143.8% enhancement in CO_2_ adsorption capacity compared to pure 6FDA-DAM membranes. Conversely, the LP-ZU-61 system displays an inverse loading-dependent trend. At 30 wt.% loading, the LP-ZU-61/6FDA-DAM membrane reduces CO_2_ adsorption capacity to 70% of the pure polymer benchmark. This divergence originates from fundamentally distinct mechanisms. Defect-engineered HP-ZU-61 generates strong CO_2_ adsorption sites through unsaturated metal centers, whereas defect-free LP-ZU-61 contains narrower micropores that restrict connectivity within polymer free-volume elements [25]. Consequently, reduced accessibility of adsorption sites contributes to the deteriorated adsorption performance of LP-ZU-61 systems. Quantitative analysis of the thermodynamic regulation of gas sorption in defect-engineered HP-ZU-61/6FDA-DAM MMMs was conducted using the dual-mode sorption model. The HP-ZU-61/6FDA-DAM membrane elevated a higher Henry’s constant, Langmuir adsorption capacity, and affinity parameter (Table S1). Conversely, the LP-ZU-61/6FDA-DAM membrane showed the inverse trend. These findings are in full agreement with the measured adsorption trends.

For ZU-61/6FDA-DAM mixed-matrix membranes (MMMs), a series of samples with filler loadings ranging from 10 to 40 wt.% were fabricated and evaluated. The membrane containing 40 wt.% ZU-61 suffered from significant mechanical failure, exhibiting extreme brittleness and a pronounced tendency to crack during drying or handling (Figure S7). Accordingly, for the HP-ZU-61/6FDA-DAM system, 30 wt.% was established as the practical upper loading limit to obtain robust, defect-free, and processable membranes.

**Figure 7:**
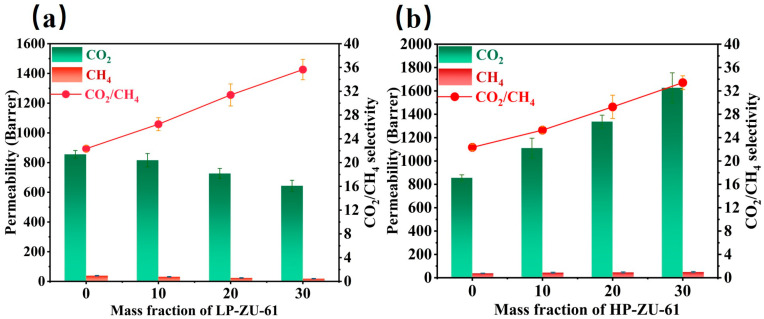
Effects of MOF filling loading on CO_2_ permeability and selectivity of membranes tested at 2 bar and 35 °C. (**a**) LP-ZU-61/6FDA-DAM membranes, (**b**) HP-ZU-61/6FDA-DAM membranes.

The gas separation performance of MMMs prepared by the defect-engineering strategy was evaluated by the Wicke–Kallenbach method using an equimolar amount of a CO_2_/CH_4_ mixture. As shown in Figure 7a,b, the average CO_2_ permeability of the pure 6FDA-DAM membrane was 855.57 barrer, and the average selectivity was 22.3, which was in agreement with the results previously reported in the literature [26,27]. For the LP-ZU-61/6FDA-DAM MMMs, the CO_2_ permeability was gradually decreased, and the selectivity was elevated with the increase in filler loading. The 30 wt.% LP-ZU-61/6FDA-DAM MMM exhibited a substantial 159.5% boost in CO_2_/CH_4_ selectivity. Nonetheless, its CO_2_ permeability (643.53 barrer) decreased to 75% of the pristine 6FDA-DAM membrane’s value (Figure 7a). This indicates that the incorporation of LP-ZU-61 nanoparticles is postulated to perturb the local chain packing of the 6FDA-DAM polymer at the filler–matrix interface. Owing to its rigid aromatic architecture, the ZU-61 framework potentially restricts the mobility of adjacent polymer chains. Consequently, this “interfacial sealing” effect mitigates non-selective gas transport through the polymer phase surrounding the filler particles. The HP-ZU-61/6FDA-DAM MMMs, with increasing filler loading, showed a significant increase in CO_2_ permeability along with enhanced selectivity. Remarkably, the 30 wt.% HP-ZU-61/6FDA-DAM membranes attained a CO_2_ permeability of 1626.3 barrer (Figure 7b). This represents a substantial increase of 252.7% relative to the 30 wt.% LP-ZU-61 system and 190% compared to the pristine 6FDA-DAM membrane. Concurrently, CO_2_/CH_4_ selectivity was enhanced by 149.8% over the polymer membrane. The concurrent enhancement of CO_2_ permeability and CO_2_/CH_4_ selectivity in the MMMs originates from a precisely tailored defect-engineering strategy, where ligand deficiencies create rapid diffusion pathways while unsaturated metal sites serve as strong CO_2_ adsorption centers [12,28].

**Figure 8:**
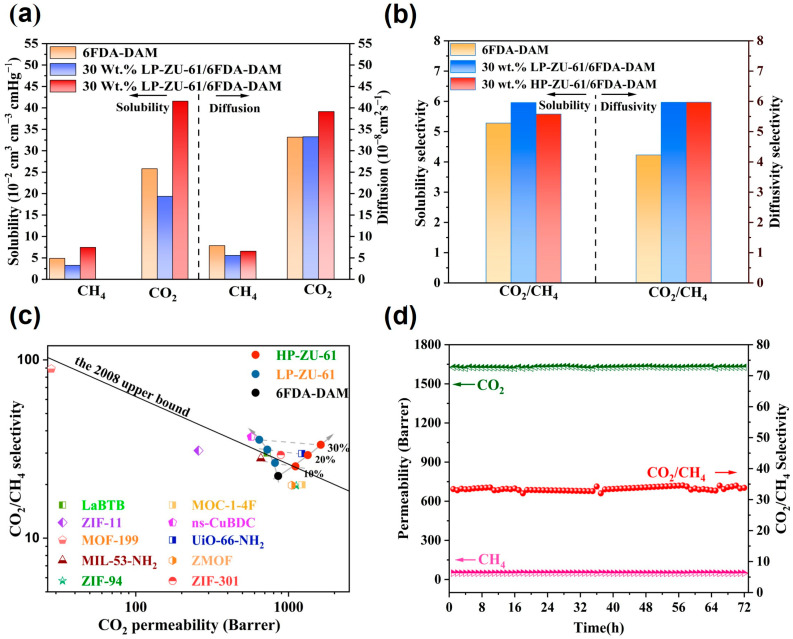
(**a**) Gas solubility and diffusivity of 6FDA-DAM membrane, 30 wt.% LP-ZU-61/6FDA-DAM membrane, and 30 wt.% HP-ZU-61/6FDA-DAM membrane; (**b**) solubility selectivity and diffusion selectivity of 6FDA-DAM membrane, 30 wt.% LP-ZU-61/6FDA-DAM membrane, and 30 wt.% HP-ZU-61/6FDA-DAM membrane; (**c**) comparison of membrane gas separation performance with other MOF/6FDA-DAM MMMs and the 2008 Robeson upper bound; (**d**) stability testing of the permeability and selectivity for CO_2_ and CH_4_ of the 30 wt.% HP-ZU-61/6FDA-DAM membranes at 2 bar and 35 °C.

Gas transport in ZU-61/6FDA-DAM MMMs was investigated via the solution-diffusion model (Figure 8a,b, Tables S1 and S2). Permeability (P) was defined as a product of the solubility coefficient (S) and the diffusion coefficient (D) [29]. Notably, the diffusion coefficients of CO_2_ of HP-ZU-61/6FDA-DAM membranes were significantly increased, and the solubility selectivity was not significantly decreased. This originated from the creation of strong Lewis acid–base adsorption sites by liganded unsaturated metal sites [28]. Dual-mode sorption modeling demonstrated increases in both the Langmuir adsorption capacity and affinity constant (Table S1), resulting in significantly enhanced CO_2_ adsorption. Notably, the CO_2_ diffusivity coefficient of the HP-ZU-61/6FDA-DAM membranes was also significantly increased, and the diffusion selectivity was slightly enhanced. This property directly maintains the separation selectivity of the HP-ZU-61/6FDA-DAM MMMs. It originates from the defect-engineered high-porosity ZU-61, which modulates the free volume elements of the 6FDA-DAM polymer and generates additional transport pathways [12]. Concurrently, the engineered pore structure establishes highly diffusive conduits, thereby reducing gas diffusion tortuosity within the 6FDA-DAM polymer matrix [4].

Figure 8c indicates that the separation performance (CO_2_ permeability: 1626.3 barrer, CO_2_/CH_4_ selectivity: 33.4) significantly surpasses the 2008 Robeson upper bound and exceeds that of most reported MOF/6FDA-DAM MMMs (Tables S3 and S4). This work demonstrates that defect engineering enables dual optimization of CO_2_ permeability and CO_2_/CH_4_ selectivity. Crucially, this work reveals two unique mechanisms: (i) ligand deficiencies in HP-ZU-61 disrupt 6FDA-DAM polymer chain packing, generating additional CO_2_ transport pathways that accelerate diffusion; (ii) unsaturated metal centers create strong Lewis acid–base adsorption sites specifically enhancing CO_2_ capture, thereby synergistically elevating separation efficiency for industrial natural gas purification.

To evaluate the long-term operational stability of the defect-engineering strategy in mixed-matrix membranes, we conducted a 72 h continuous CO_2_/CH_4_ separation test at 35 °C and 2 bar for both 30 wt.% LP-ZU-61/6FDA-DAM (Figure S8) and HP-ZU-61/6FDA-DAM membranes (Figure 8d). The results demonstrate that two membrane types maintained their initial CO_2_ permeability and CO_2_/CH_4_ selectivity throughout the testing period. This confirms that the HP-ZU-61/6FDA-DAM membranes fabricated through defect engineering achieve both high flux and selectivity without compromising their long-term operational stability, providing a practical solution for industrial-scale natural gas purification membranes.

## 4. Conclusions

In summary, this work demonstrates that defect-engineered MOFs offer an effective strategy for enhancing the gas separation performance of MOF/polymer mixed-matrix membranes. Material characterization confirmed that the high-porosity HP-ZU-61 filler exhibits morphology, size, and membrane interfacial properties comparable to those of its pristine counterpart, establishing a well-defined system for investigating gas transport mechanisms in MMMs. The pyridine modulator-induced defects drive dual performance enhancement: CO_2_ permeability surges to 1626.3 barrer with CO_2_/CH_4_ selectivity reaching 33.4 at 30 wt.% loading, collectively surpassing the 2008 Robeson upper bound. Crucially, solution-diffusion modeling attributes this enhancement to unsaturated metal sites in HP-ZU-61 boosting CO_2_ adsorption alongside ligand deficiencies accelerating diffusion pathways. The validated strategy demonstrates that rational modification of existing MOFs outperforms conventional new MOF development approaches for MMM optimization.

## Data Availability

The original contributions presented in this study are included in the article and the Supplementary Materials. Further inquiries can be directed to the corresponding author.

## Supplementary Materials

The following supporting information can be downloaded at: https://www.mdpi.com/article/10.3390/membranes15100296/s1, Figure S1: Schematic diagram for gas permeation test; Figure S2: SEM images of ZU-61 particles with varying H_2_O/ethylene glycol ratios; Figure S3: XRD patterns of ZU-61 with varying H_2_O/ethylene glycol ratios; Figure S4: (a) FTIR of HP-ZU-61 and LP-ZU-61; (b) TGA of HP-ZU-61 and LP-ZU-61; Figure S5: (a) XRD patterns of LP-ZU-61 particles and ZU-61 particles with varying concentrations of pyridine modulator; (b) N_2_ sorption isotherms of LP-ZU-61 particles and ZU-61 particles with varying concentrations of pyridine modulator at 77K; Figure S6: Pore size distributions of HP-ZU-61 and LP-ZU-61 by the DFT approach; Figure S7: The images of membranes ((left) 40 wt.% LP-ZU-61/6FDA-DAM membrane; (right) 40 wt.% LP-ZU-61/6FDA-DAM membrane); Figure S8: Stability testing of the permeability and selectivity for CO_2_ and CH_4_ of the 30 wt.% HP-ZU-61/6FDA-DAM membranes at 2 bar and 35 °C; Table S1: Dual-mode sorption parameters and solubility coefficients of ZU-61/6FDA-DAM MMMs determined from their sorption isotherms for CO_2_ and CH_4_; Table S2: Diffusivity coefficients, solubility coefficients, and permeability coefficients of defect-engineered ZU-61/6FDA-DAM MMMs for CO_2_ and CH_4_ determined from sorption and permeability data at 2 bar and 35 °C; Table S3: The thickness and CO_2_/CH_4_ separation performance of ZU-61-based MMMs in this work; Table S4: Comparison of the CO_2_/CH_4_ separation performance of ZU-61-based MMMs with other mixed matrixes. References [23,30,31,32,33,34,35,36,37] are cited in the Supplementary Materials.

## References

[1] Gajanan K., Ranjith P.G., Yang S.Q., Xu T. (2024). Advances in Research and Developments on Natural Gas Hydrate Extraction with Gas Exchange. Renew. Sustain. Energy Rev..

[2] Zhang Y., Jangodaz E., Yin B.H., Telfer S.G. (2024). Functionalisation of MUF-15 Enhances CO_2_/CH_4_ Selectivity in Mixed-Matrix Membranes. Chem. Commun..

[3] Koros W.J., Zhang C. (2017). Materials for Next-Generation Molecularly Selective Synthetic Membranes. Nat. Mater..

[4] Yan J., Sun Y., Ji T., Wu M., Meng S., Dong W., Liu Y., Yu K., Hu W., Sun B. (2024). Enhanced CO2/N2 Separation in Hybrid Composite Membrane via Dispersion of Hollow Defect-engineered Zr-MOF Nanoparticles. AIChE J..

[5] Hou R., Xie J., Gu Y., Wang L., Pan Y. (2025). Simultaneously Enhanced Permeability and Selectivity of Pebax-1074-Based Mixed-Matrix Membrane for CO_2_ Separation. Membranes.

[6] Lee T.H., Roh J.S., Yoo S.Y., Roh J.M., Choi T.H., Park H.B. (2020). High-Performance Polyamide Thin-Film Nanocomposite Membranes Containing ZIF-8/CNT Hybrid Nanofillers for Reverse Osmosis Desalination. Ind. Eng. Chem. Res..

[7] Knebel A., Bavykina A., Datta S.J., Sundermann L., Garzon-Tovar L., Lebedev Y., Durini S., Ahmad R., Kozlov S.M., Shterk G. (2020). Solution Processable Metal–Organic Frameworks for Mixed Matrix Membranes Using Porous Liquids. Nat. Mater..

[8] Lee T.H., Lee B.K., Youn C., Kang J.H., Kim Y.J., Kim K.I., Ha Y.R., Han Y., Park H.B. (2023). Interface Engineering in MOF/Crosslinked Polyimide Mixed Matrix Membranes for Enhanced Propylene/Propane Separation Performance and Plasticization Resistance. J. Membr. Sci..

[9] Wang H., He S., Qin X., Li C., Li T. (2018). Interfacial Engineering in Metal–Organic Framework-Based Mixed Matrix Membranes Using Covalently Grafted Polyimide Brushes. J. Am. Chem. Soc..

[10] Li C., Liu J., Zhang K., Zhang S., Lee Y., Li T. (2021). Coating the Right Polymer: Achieving Ideal Metal–Organic Framework Particle Dispersibility in Polymer Matrixes Using a Coordinative Crosslinking Surface Modification Method. Angew. Chem. Int. Ed..

[11] Cheng Y., Ying Y., Japip S., Jiang S., Chung T., Zhang S., Zhao D. (2018). Advanced Porous Materials in Mixed Matrix Membranes. Adv. Mater..

[12] Li S., Han W., An Q., Yong K., Yin M. (2023). Defect Engineering of MOF-Based Membrane for Gas Separation. Adv. Funct. Mater..

[13] Teesdale J.J., Lee M., Lu R., Smith Z.P. (2023). Uncertainty in Composite Membranes: From Defect Engineering to Film Processing. J. Am. Chem. Soc..

[14] Cui X., Niu Z., Shan C., Yang L., Hu J., Wang Q., Lan P.C., Li Y., Wojtas L., Ma S. (2020). Efficient Separation of Xylene Isomers by a Guest-Responsive Metal–Organic Framework with Rotational Anionic Sites. Nat. Commun..

[15] Zornoza B., Tellez C., Coronas J., Gascon J., Kapteijn F. (2013). Metal Organic Framework Based Mixed Matrix Membranes: An Increasingly Important Field of Research with a Large Application Potential. Micropor. Mesopor. Mater..

[16] Zhuang Z., Cheng J., Wang X., Zhao B., Han X., Luo Y. (2007). Surface-Enhanced Raman Spectroscopy and Density Functional Theory Study on 4,4′-Bipyridine Molecule. Spectrochim. Acta Part A Mol. Biomol. Spectrosc..

[17] Lv J., Cui Y., Yang J., Li L., Zhou X., Lu J., He G. (2022). Inorganic Pillar Center-Facilitated Counterdiffusion Synthesis for Highly H_2_ Perm-Selective KAUST-7 Membranes. ACS Appl. Mater. Interfaces.

[18] Lee T.H., Ozcan A., Park I., Fan D., Jang J.K., Mileo P.G.M., Yoo S.Y., Roh J.S., Kang J.H., Lee B.K. (2021). Disclosing the Role of Defect-Engineered Metal–Organic Frameworks in Mixed Matrix Membranes for Efficient CO_2_ Separation: A Joint Experimental-Computational Exploration. Adv. Funct. Mater..

[19] Zhu B., He S., Yang Y., Li S., Lau C.H., Liu S., Shao L. (2023). Boosting Membrane Carbon Capture via Multifaceted Polyphenol-Mediated Soldering. Nat. Commun..

[20] Qiu W., Xu L., Chen C.-C., Paul D.R., Koros W.J. (2013). Gas Separation Performance of 6FDA-Based Polyimides with Different Chemical Structures. Polymer.

[21] Chen K., Xu K., Xiang L., Dong X., Han Y., Wang C., Sun L.-B., Pan Y. (2018). Enhanced CO_2_/CH_4_ Separation Performance of Mixed-Matrix Membranes through Dispersion of Sorption-Selective MOF Nanocrystals. J. Membr. Sci..

[22] Rodenas T., Van Dalen M., García-Pérez E., Serra-Crespo P., Zornoza B., Kapteijn F., Gascon J. (2014). Visualizing MOF Mixed Matrix Membranes at the Nanoscale: Towards Structure-Performance Relationships in CO_2_/CH_4_ Separation Over NH_2_-MIL-53(Al)@PI. Adv. Funct. Mater..

[23] Safak Boroglu M., Yumru A.B. (2017). Gas Separation Performance of 6FDA-DAM-ZIF-11 Mixed-Matrix Membranes for H_2_/CH_4_ and CO_2_/CH_4_ Separation. Sep. Purif. Technol..

[24] Yu B., Lan T., Wang H., Han L., Liu Y., Li J., Li L. (2025). Reversed CO2/C2H2 Separation via Combined Adsorption Kinetics and Diffusion Differences in MOF-Polymer Mixed Matrix Membrane. J. Membr. Sci..

[25] Shang Q., Liu Y., You Q., Yan Y., Yang X., Liao G., Wang D. (2024). Introduction of Mesopores Effectively Enhances the Accessibility of Volatile Organic Compounds within the Micropores of Covalent Triazine Frameworks. Chem. Eng. J..

[26] Yang X., Wang Y., Hua J., Hou R., Chen J., Gong Q., Wang C., Pan Y. (2024). Co-Fa Nanoplates Incorporated 6FDA-DAM Mixed-Matrix Membranes for Enhanced CO_2_/CH_4_ Separation. Sep. Purif. Technol..

[27] Bachman J.E., Long J.R. (2016). Plasticization-Resistant Ni_2_ (Dobdc)/Polyimide Composite Membranes for the Removal of CO_2_ from Natural Gas. Energy Environ. Sci..

[28] Lee T.H., Jung J.G., Kim Y.J., Roh J.S., Yoon H.W., Ghanem B.S., Kim H.W., Cho Y.H., Pinnau I., Park H.B. (2021). Defect Engineering in Metal–Organic Frameworks Towards Advanced Mixed Matrix Membranes for Efficient Propylene/Propane Separation. Angew. Chem. Int. Ed..

[29] Liu T., Zhang R., Huang G., Xie Y., Xie L.-H., Li J.-R. (2023). Mixed Matrix Membranes Based on Soluble Perfluorinated Metal-Organic Cage and Polyimide for CO_2_/CH_4_ Separation. Sep. Purif. Technol..

[30] Wang Z., Yuan J., Li R., Zhu H., Duan J., Guo Y., Liu G., Jin W. (2021). ZIF-301 MOF/6FDA-DAM Polyimide Mixed-Matrix Membranes for CO_2_/CH_4_ Separation. Sep. Purif. Technol..

[31] Ahmad M.Z., Navarro M., Lhotka M., Zornoza B., Téllez C., De Vos W.M., Benes N.E., Konnertz N.M., Visser T., Semino R. (2018). Enhanced Gas Separation Performance of 6FDA-DAM Based Mixed Matrix Membranes by Incorporating MOF UiO-66 and Its Derivatives. J. Membr. Sci..

[32] Liu G., Labreche Y., Chernikova V., Shekhah O., Zhang C., Belmabkhout Y., Eddaoudi M., Koros W.J. (2018). Zeolite-like MOF Nanocrystals Incorporated 6FDA-Polyimide Mixed-Matrix Membranes for CO_2_/CH_4_ Separation. J. Membr. Sci..

[33] Hua Y., Wang H., Li Q., Chen G., Liu G., Duan J., Jin W. (2018). Highly Efficient CH_4_ Purification by LaBTB PCP-Based Mixed Matrix Membranes. J. Mater. Chem. A.

[34] Nuhnen A., Klopotowski M., Tanh Jeazet H.B., Sorribas S., Zornoza B., Téllez C., Coronas J., Janiak C. (2020). High Performance MIL-101(Cr)@6FDA-*m*PD and MOF-199@6FDA-*m*PD Mixed-Matrix Membranes for CO_2_/CH_4_ Separation. Dalton Trans..

[35] Sabetghadam A., Seoane B., Keskin D., Duim N., Rodenas T., Shahid S., Sorribas S., Guillouzer C.L., Clet G., Tellez C. (2016). Metal Organic Framework Crystals in Mixed-Matrix Membranes: Impact of the Filler Morphology on the Gas Separation Performance. Adv. Funct. Mater..

[36] Etxeberria-Benavides M., David O., Johnson T., Łozińska M.M., Orsi A., Wright P.A., Mastel S., Hillenbrand R., Kapteijn F., Gascon J. (2018). High Performance Mixed Matrix Membranes (MMMs) Composed of ZIF-94 Filler and 6FDA-DAM Polymer. J. Membr. Sci..

[37] Yang Y., Goh K., Wang R., Bae T.-H. (2017). High-Performance Nanocomposite Membranes Realized by Efficient Molecular Sieving with CuBDC Nanosheets. Chem. Commun..

