# Supplementary material for: Defect Engineering in Fluorinated Metal–Organic Frameworks Within Mixed-Matrix Membranes for Enhanced CO2 Separation
Source: Membranes (Basel). 2025 Sep 30;15(10):296. doi: 10.3390/membranes15100296 (PMC12565906; doi:10.3390/membranes15100296)
Supplement: Supplementary file 1 [file membranes-15-00296-s001.zip › membranes-3852423-supplementary.pdf]

Supplementary Information

Article

Defect Engineering in Fluorinated Metal–Organic Frameworks within Mixed-Matrix Membranes for Enhanced CO_2 Separation

Benxing Li[1], Lei Wang[1], Yizheng Tao[1], Rujing Hou[1] Faheem Hassan Akhtar[2] and Yichang Pan[*1]

1 State Key Laboratory of Materials-Oriented Chemical Engineering, College of chemical Engineering, Nanjing Tech University, Nanjing 210009, China. 2 Lahore University of Management Sciences (LUMS)

202461104013@njtech.edu.cn (B.L.); 202362042066@njtech.edu.cn (L.W.); 202361204444@njtech.edu.cn (Y.T.); rujing.hou@njtech.edu.cn (R.H.); faheem.akhtar@lums.edu.pk (F.A).

*Corresponding author. E-mail: panyc@njtech.edu.cn (Y. P.)

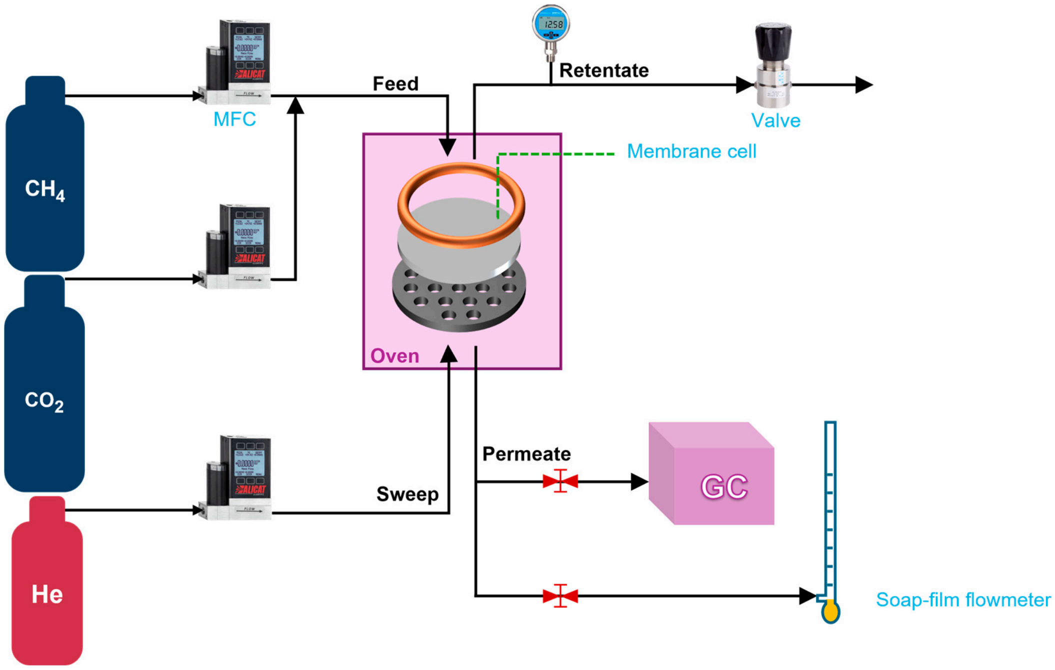

Figure S1. Schematic diagram for gas permeation test.

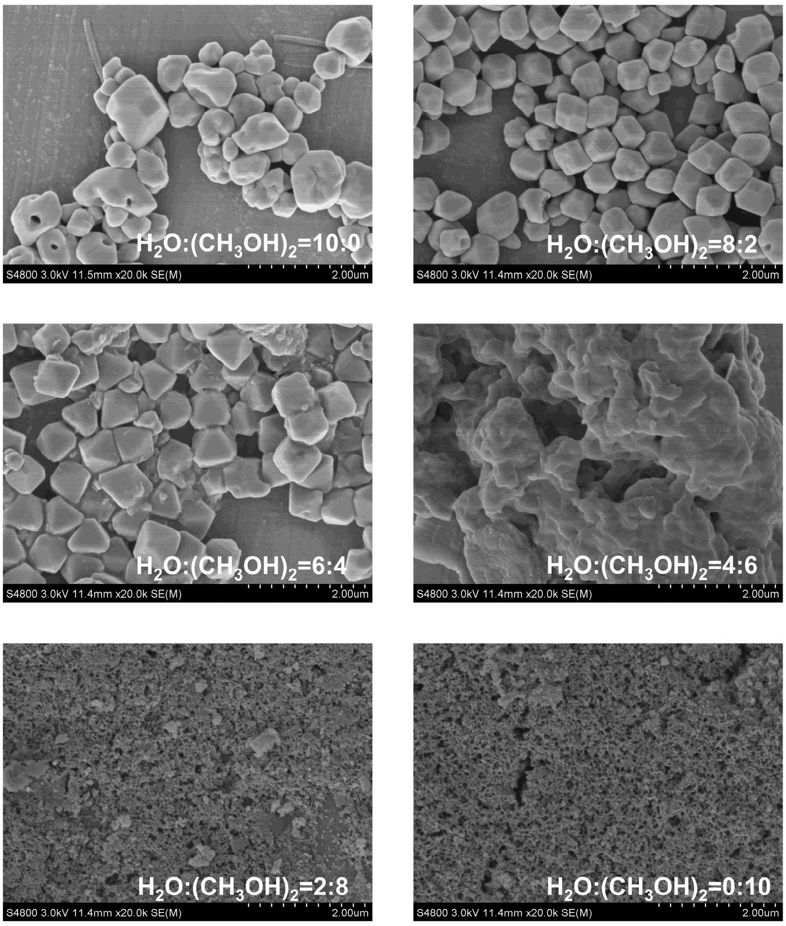

Figure S2. SEM images of ZU-61 particles with varying H_2O/ethylene glycol ratios.

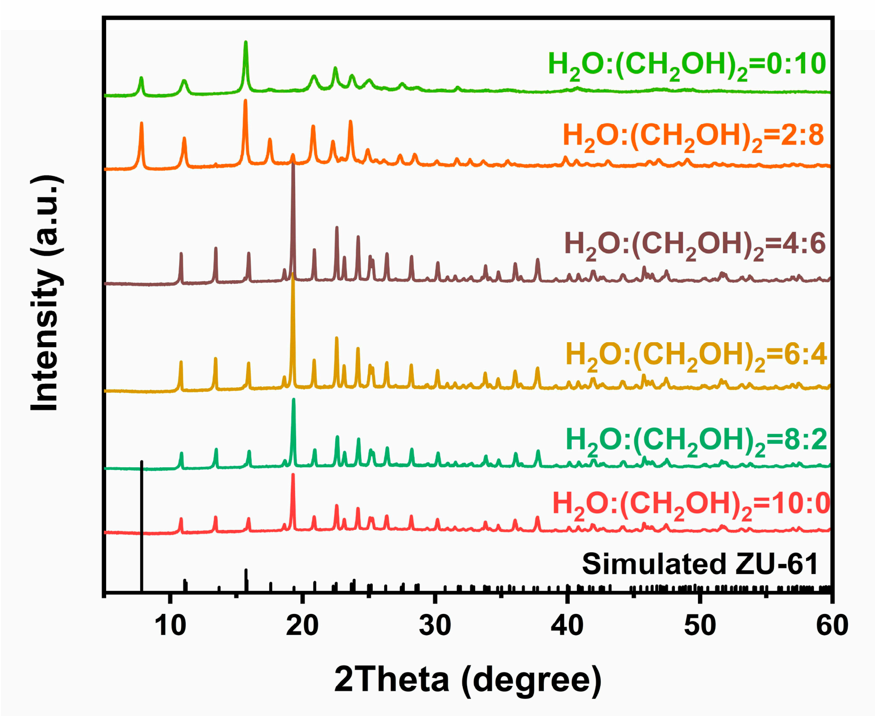

Figure S3. XRD patterns of ZU-61 with varying H_2O/ethylene glycol ratios.

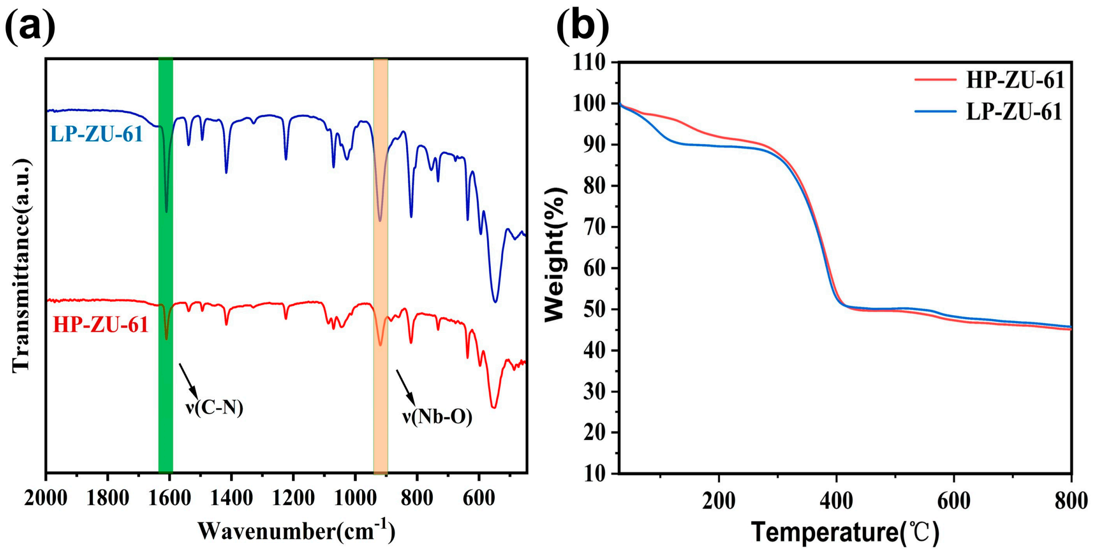

Figure S4. (a) FTIR of HP-ZU-61 and LP-ZU-61. (b) TGA of HP-ZU-61 and LP-ZU-61.

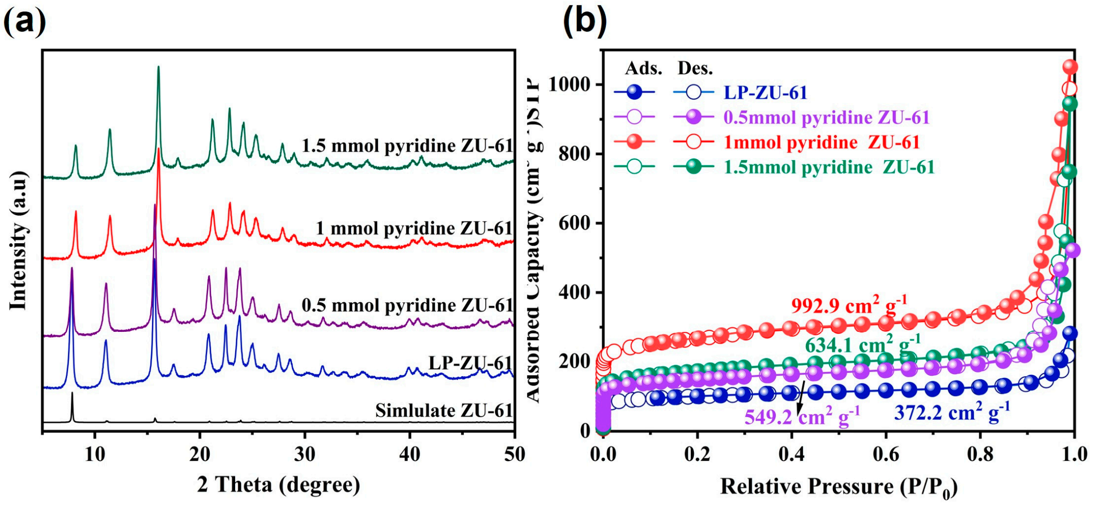

Figure S5. (a) XRD patterns of LP-ZU-61 particles and ZU-61 particles with varying concentrations of pyridine modulator; (b) N_2 sorption isotherms of LP-ZU-61 particles and ZU-61 particles with varying concentrations of pyridine modulator at 77K.

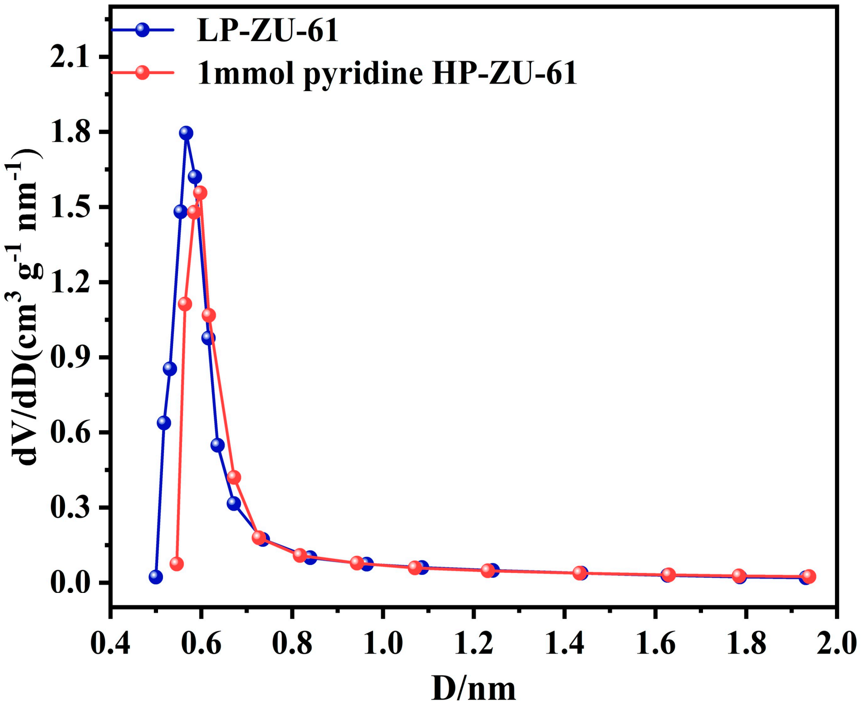

Figure S6. Pore size distributions of HP-ZU-61 and LP-ZU-61 by the DFT approach.

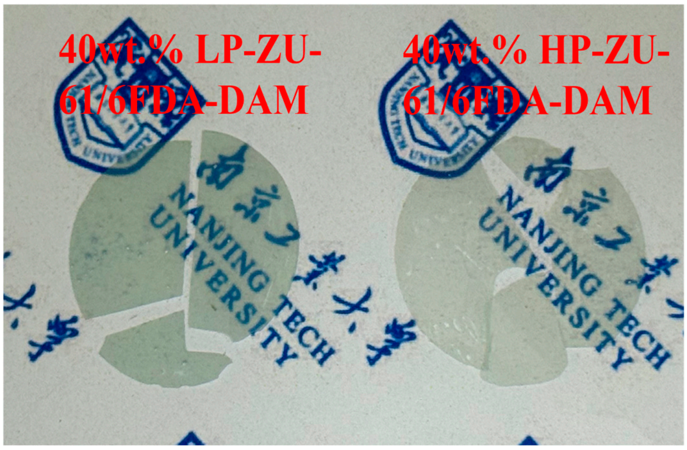

Figure S7. The images of membranes ((left) 40 wt.% LP-ZU-61/6FDA-DAM membrane; (right) 40 wt.% LP-ZU-61/6FDA-DAM membrane).

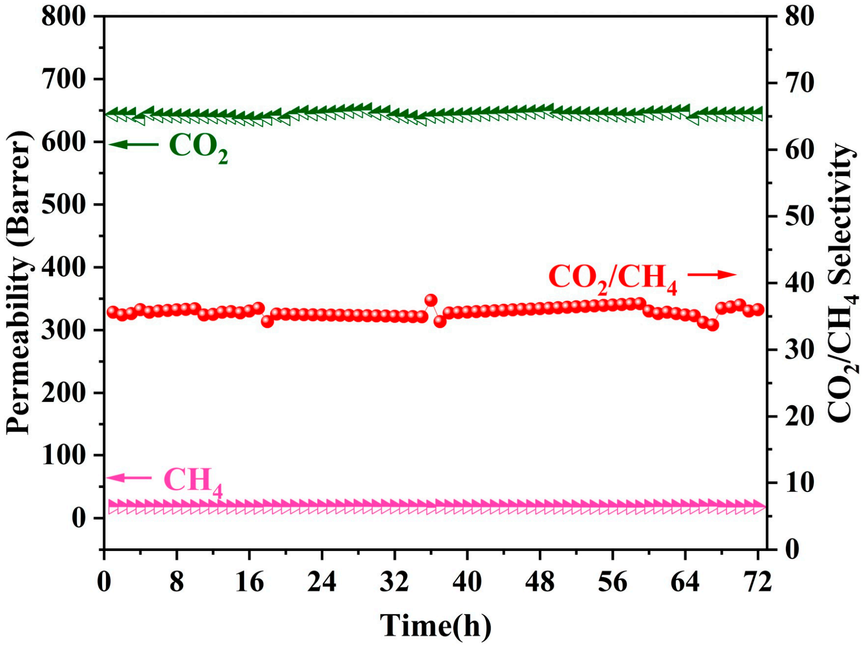

Figure S8. Stability testing of the permeability and selectivity for CO_2 and CH_4 of the 30 wt.% HP-ZU-61/6FDA-DAM membranes at 2 bar and 35 °C.

Table S1. Dual-mode sorption parameters and solubility coefficients of ZU-61/6FDA-DAM MMMs determined from their sorption isotherms for CO_2 and CH_4.

Membranes	k_d ($cm^3(STP)\cdot cm^{-3}\cdot cmHg^{-1}$)		c'_h ($cm^3(STP)\cdot cm^{-3}$)		b ($cmHg^{-1}$)		Solubility ($cm^3(STP)\cdot cm^{-3}\cdot cmHg^{-1}$)	
	CO_2	CH_4	CO_2	CH_4	CO_2	CH_4	CO_2	CH_4
6FDA-DAM	10.93	3.62	22.47	2.59	1.95	0.94	25.80	4.89
HP-ZU-61/6FDA-DAM	18.34	5.522	33.48	3.95	2.26	0.95	41.56	7.445
LP-ZU-61/6FDA-DAM	9.57	2.38	13.08	1.6	2.95	1.18	19.35	3.26

Table S2. Diffusivity coefficients, solubility coefficients, and permeability coefficients of defect-engineered ZU-61/6FDA-DAM MMMs for CO_2 and CH_4 determined from sorption and permeability data at 2 bar and 35 °C.

Membranes	Solubility coefficients (cm^3(STP)·cm^{3}·$cmHg^{-1}$)			Diffusivity coefficient ($10^{-8}cm^2s^{-1}$)			Permeability (P = D × S, barrer)		
	S_{CO2}	S_{CH4}	$S_{CO2/CH4}$	D_{CO2}	D_{CH4}	$D_{CO2/CH4}$	P_{CO2}	P_{CH4}	$\alpha_{CO2/CH4}$
6FDA-DAM	25.80	4.89	5.28	33.16	7.85	4.23	855.57	38.33	22.34
HP-ZU-61/6FDA-DAM	41.56	7.445	5.58	39.13	6.55	5.97	1626.33	48.80	33.41
LP-ZU-61/6FDA-DAM	19.35	3.26	5.96	33.25	5.57	5.96	643.53	18.10	35.64

Table S3. The thickness and CO_2/CH_4 separation performance of ZU-61-based MMMs in this work.

Membrane	Thickness	Loading (wt.%)	T/P (°C/bar)	P_{CO2} (barrer)	$\alpha_{(CO2/CH4)}$
6FDA-DAM	50.8±2.2μm	0	35/2	855.57±25.77	22.3±0.53
HP-ZU-61/6FDA-DAM	55.04±1.21μm	10	35/2	1110.70±84.48	25.29±0.56
HP-ZU-61/6FDA-DAM	59.25±2μm	20	35/2	1336.83±55.62	29.26±1.99
HP-ZU-61/6FDA-DAM	63.38±1.8μm	30	35/2	1626.33±128.46	33.41±1.17
LP-ZU-61/6FDA-DAM	54.12±1.9μm	10	35/2	815.83±44.6	26.45±1.08
LP-ZU-61/6FDA-DAM	57.85±2.2μm	20	35/2	726.6±34	35.6±1.85
LP-ZU-61/6FDA-DAM	60.96±2.1μm	20	35/2	643.53±37.58	35.6±1.71

Table S4. Comparison of the CO_2/CH_4 separation performance of ZU-61-based MMMs with other mixed matrixes.

Membrane	Loading (wt.%)	T/P (°C/bar)	P_{CO2} (barrer)	$\alpha_{(CO2/CH4)}$	Reference
ZIF-301/6FDA-DAM	20	25/4	891	29.3	[1]
UiO-66-NH_2/6FDA-DAM	16	35/2	1223	29.8	[2]
ZMOF/6FDA-DAM	20	35/3.45	1050	19.8	[3]
LABTB/6FDA-DAM	10	25/3.5	725	30	[4]
ZIF-11/6FDA-DAM	20	30/4	257.5	31	[5]
MOF-199/6FDA-DAM	24	35/3	28	89	[6]
MIL-53-NH_2/6FDA-DAM	20	25/3	659.73	28.00	[7]
ZIF-94/6FDA-DAM	30	25/2	1125	19.7	[8]
ns-CuBDC/6FDA-DAM	2	25/1	570	37	[9]
MOC-1-4F/6FDA-DAM	2.17	35/3	1228.4	19.9	[10]

Membranes measured under similar conditions.

Reference:

1. Wang, Z.; Yuan, J.; Li, R.; Zhu, H.; Duan, J.; Guo, Y.; Liu, G.; Jin, W. ZIF-301 MOF/6FDA-DAM Polyimide Mixed-Matrix Membranes for CO2/CH4 Separation. *Sep. Purif. Technol.* **2021**, *264*, 118431, doi:10.1016/j.seppur.2021.118431.
2. Ahmad, M.Z.; Navarro, M.; Lhotka, M.; Zornoza, B.; Téllez, C.; De Vos, W.M.; Benes, N.E.; Konnertz, N.M.; Visser, T.; Semino, R.; et al. Enhanced Gas Separation Performance of 6FDA-DAM Based Mixed Matrix Membranes by Incorporating MOF UiO-66 and Its Derivatives. *J. Membr. Sci.* **2018**, *558*, 64–77, doi:10.1016/j.memsci.2018.04.040.
3. Liu, G.; Labreche, Y.; Chernikova, V.; Shekhah, O.; Zhang, C.; Belmabkhout, Y.; Eddaoudi, M.; Koros, W.J. Zeolite-like MOF Nanocrystals Incorporated 6FDA-Polyimide Mixed-Matrix Membranes for CO2/CH4 Separation. *J. Membr. Sci.* **2018**, *565*, 186–193, doi:10.1016/j.memsci.2018.08.031.
4. Hua, Y.; Wang, H.; Li, Q.; Chen, G.; Liu, G.; Duan, J.; Jin, W. Highly Efficient CH_4 Purification by LaBTB PCP-Based Mixed Matrix Membranes. *J. Mater. Chem. A* **2018**, *6*, 599–606, doi:10.1039/c7ta07261a.
5. Safak Boroglu, M.; Yumru, A.B. Gas Separation Performance of 6FDA-DAM-ZIF-11 Mixed-Matrix Membranes for H2/CH4 and CO2/CH4 Separation. *Sep. Purif. Technol.* **2017**, *173*, 269–279, doi:10.1016/j.seppur.2016.09.037.
6. Nuhnen, A.; Klopotowski, M.; Tanh Jeazet, H.B.; Sorribas, S.; Zornoza, B.; Téllez, C.; Coronas, J.; Janiak, C. High Performance MIL-101(Cr)@6FDA-*m*PD and MOF-199@6FDA-*m*PD Mixed-Matrix Membranes for CO_2/CH_4 Separation. *Dalton Trans.* **2020**, *49*, 1822–1829, doi:10.1039/c9dt03222c.
7. Sabetghadam, A.; Seoane, B.; Keskin, D.; Duim, N.; Rodenas, T.; Shahid, S.; Sorribas, S.; Guillouzer, C.L.; Clet, G.; Tellez, C.; et al. Metal Organic Framework Crystals in Mixed-Matrix Membranes: Impact of the Filler Morphology on the Gas Separation Performance. *Adv. Funct. Mater.* **2016**, *26*, 3154–3163, doi:10.1002/adfm.201505352.

8. Etxeberria-Benavides, M.; David, O.; Johnson, T.; Łozińska, M.M.; Orsi, A.; Wright, P.A.; Mastel, S.; Hillenbrand, R.; Kapteijn, F.; Gascon, J. High Performance Mixed Matrix Membranes (MMMs) Composed of ZIF-94 Filler and 6FDA-DAM Polymer. *J. Membr. Sci.* **2018**, *550*, 198–207, doi:10.1016/j.memsci.2017.12.033.
9. Yang, Y.; Goh, K.; Wang, R.; Bae, T.-H. High-Performance Nanocomposite Membranes Realized by Efficient Molecular Sieving with CuBDC Nanosheets. *Chem. Commun.* **2017**, *53*, 4254–4257, doi:10.1039/c7cc00295e.
10. Liu, T.; Zhang, R.; Huang, G.; Xie, Y.; Xie, L.-H.; Li, J.-R. Mixed Matrix Membranes Based on Soluble Perfluorinated Metal-Organic Cage and Polyimide for CO2/CH4 Separation. *Sep. Purif. Technol.* **2023**, *318*, 124006, doi:10.1016/j.seppur.2023.124006.
